# Influence of Enteric Infections on Response to Oral Poliovirus Vaccine: A Systematic Review and Meta-analysis

**DOI:** 10.1093/infdis/jiu182

**Published:** 2014-03-31

**Authors:** Edward P. K. Parker, Beate Kampmann, Gagandeep Kang, Nicholas C. Grassly

**Affiliations:** 1 Department of Infectious Disease Epidemiology; 2 Department of Paediatrics, St Mary's Campus, Imperial College London, United Kingdom; 3 MRC Unit, The Gambia, Fajara; 4 Division of Gastrointestinal Sciences, Christian Medical College, Vellore, India

**Keywords:** diarrhea, enterovirus, immunogenicity, interference, oral poliovirus vaccine

## Abstract

***Background.*** The impaired immunogenicity of oral poliovirus vaccine (OPV) in low-income countries has been apparent since the early field trials of this vaccine. Infection with enteropathogens at the time of vaccination may contribute to this phenomenon. However, the relative influence of these infections on OPV performance remains uncertain.

***Methods.*** We conducted a systematic review to examine the impact of concurrent enteric infections on OPV response. Using random-effects models, we assessed the effects of nonpolio enteroviruses (NPEVs) and diarrhea on the odds of seroconversion and/or vaccine virus shedding.

***Results.*** We identified 25 trials in which OPV outcomes were compared according to the presence or absence of enteric infections, the majority of which (n = 17) reported only on NPEVs. Concurrent NPEVs significantly reduced the odds of per-dose seroconversion for type 1 poliovirus (odds ratio [OR] 0.44, 95% confidence interval 0.23−0.84), but not type 2 (OR 0.53 [0.19−1.46]) or type 3 (OR 0.56 [0.27−1.12]). A similar reduction, significant for type 1 poliovirus (OR 0.50 [0.28−0.89]), was observed in the odds of vaccine virus shedding among NPEV-infected individuals. Concurrent diarrhea significantly inhibited per-dose seroconversion overall (OR 0.61 [0.38−0.87]).

***Conclusions.*** Our findings are consistent with an inhibitory effect of concurrent enteric infections on OPV response.

In 1988, the World Health Assembly committed to the task of eradicating polio worldwide. The ensuing efforts of the Global Polio Eradication Initiative have brought about a decline in the annual incidence of paralytic poliomyelitis by more than 99%. Yet in spite of this progress, polio remains endemic in 3 countries—Afghanistan, Pakistan, and Nigeria—and the risk of renewed transmission in countries previously clear of the disease remains.

Although the persistence of polio in the face of concerted eradication efforts cannot be attributed to any single cause, the reduced immunogenicity of oral poliovirus vaccine (OPV) in low-income countries has contributed to the disease's resilience. To date, OPV has been the vaccine of choice for eradication efforts across much of the globe on account of its ease of administration, low cost, and ability to induce mucosal immunity. However, concerns over the impaired immunogenicity of OPV in low-income countries (particularly those in tropical and subtropical regions) have existed since the earliest field trials of this vaccine [1]. In a review of studies conducted in resource-limited settings, Patriarca et al [2] documented average seroconversion rates after 3 doses of trivalent OPV (tOPV) of 73% for type 1 poliovirus (range, 36%−99%), 90% for type 2 (range, 71%−100%), and 70% for type 3 (range, 40%−100%). Corresponding rates in high-income settings typically exceed 95% for all serotypes [3].

Continued use of OPV in routine vaccination, supplementary immunization activities, and outbreak response is essential to the polio eradication endgame [4]. The diminished immunogenicity of this vaccine in low-income countries therefore represents an important public health concern. Moreover, this phenomenon is not unique to polio: oral vaccines against cholera [5] and rotavirus [6] have also demonstrated impaired immunogenicity in low-income settings.

A range of factors have been implicated in the compromised performance of oral vaccines in low-income countries, including the high incidence of enteric infections, malnutrition, diminished vaccine potency, and interference by maternal antibodies [2, 7]. The potential role of enteric infections was one of the first to be highlighted as a possible source of interference with OPV [8]. However, studies of this phenomenon have yielded contradictory results: while several reports have supported the interfering effects of concurrent nonpolio enteroviruses (NPEVs) [9, 10], bacteria [11], or diarrhea [12], others have refuted these effects [13–15]. The relative contribution of enteric infections to the impaired immunogenicity of OPV in low-income countries thus remains uncertain.

We carried out a systematic review and meta-analysis with the aim of estimating the influence of enteric infections and diarrhea on the odds of responding to OPV. The review also considered the effects of environmental enteropathy—a subclinical disorder associated with blunted intestinal villi, nutrient malabsorption, and intestinal inflammation [16]—which has been widely documented among individuals in low-income countries, and suggested as a possible cause of impaired oral vaccine performance [7, 16].

## METHODS

### Literature Search

We carried out a search of the citation databases PubMed and ISI Web of Knowledge in October 2012 (see Supplementary Materials Section 1.1 for search term). Following the removal of duplicates, titles and abstracts were evaluated for their relevance to the review topic (Supplementary Materials Section 1.2), and full-text copies of eligible articles obtained. Additional articles were identified by screening the text and bibliographies of relevant articles and conference proceedings (Supplementary Materials Section 1.3). PRISMA guidelines were followed throughout the study selection process [17].

Studies were included in the review if they fulfilled the following criteria: (1) delivery of OPV in a prospective trial; (2) reporting 0–7 days prior to vaccination of diarrhea, NPEV excretion, other indicators of enteric coinfection, or markers of environmental enteropathy; and (3) measurement of OPV response, including assessment of seroconversion or polio-specific antibody titers within 8 weeks of vaccination, shedding of OPV between 1 and 4 weeks after vaccination, or intestinal immunity (including measurement of polio-specific fecal immunoglobin A following vaccination or shedding of vaccine virus after OPV challenge). If the reporting of enteric infections spanned the 7 days preceding vaccination, but was not limited to this window, the study was included. Studies were excluded if: (1) they included only immunocompromised individuals; (2) less than 10 subjects were present in the infected or control group; (3) OPV outcomes were not presented according to the presence or absence of concurrent infection, diarrhea, or markers of environmental enteropathy; or (4) the reporting of enteroviruses did not distinguish polioviruses from NPEVs. Conference abstracts were excluded if a complete report of the study was already included in our review. If multiple eligible reports of the same trial were encountered, the most comprehensive report was used for data extraction. Several reports presented data from separate trials of monovalent OPV (mOPV) and tOPV [18, 19], or of tOPV trials conducted in different countries [20]—these were considered as separate studies during the analysis. Publications in languages other than English were translated with the assistance of proficient speakers.

### Data Extraction

Data were extracted from eligible studies regarding the type, schedule, and potency of the administered OPV, the timing of sample collection, the collection method for fecal specimens, the laboratory methods used for the assessment of enteric viruses, the criteria used to define diarrhea, and the criteria and methods used to assess serological response. Results presented in graphical form were digitized and data extracted using Plot Digitizer software [21]. Where relevant, an effort was made to obtain supplementary details from authors of the included studies.

### Statistical Analysis

We carried out a meta-analysis to examine the effects of concurrent NPEVs and diarrhea on the odds of seroconversion per dose of OPV, and of concurrent NPEVs on the odds of vaccine virus shedding (intestinal immunity was not used to assess OPV response in any of the eligible studies). Studies were included in the analysis if they reported serotype-specific seroconversion or vaccine virus shedding after a given dose of OPV according to the presence or absence of NPEVs (excluding poliovirus-infected individuals) or diarrhea. If dose-specific outcomes were reported for multiple OPV doses within a trial, data for the first administered dose were used. An additional analysis was conducted to compare the odds of seroconversion after multiple OPV doses in individuals presenting with diarrhea at the time of 1 or more doses with those free of diarrhea at all doses. If data were presented as proportions, the number of responders was inferred by rounding to the nearest whole number. Summary odds ratios (ORs) and 95% confidence intervals (CIs) were calculated separately for each serotype on a log scale using random-effects models [22]. Heterogeneity among studies was assessed using the χ^2^ statistic. A continuity correction of 0.5 was used in trials with zero events (zero responders or nonresponders) in 1 or both study arms. Data across the serotypes were then combined in a multilevel meta-analytic model based on structural equation modeling [23], incorporating study as a cluster effect. Overall ORs for the multilevel model were calculated using maximum likelihood estimation, with likelihood-based CIs. Mixed-effects meta-regression analyses were used to assess the impact of serotype, formulation (mOPV vs tOPV), and trial setting (low-, lower-middle- or upper-middle-income vs high-income countries, as currently listed by the World Bank [24]) on effect size; the impact of each factor on the fit of the model was assessed using the likelihood ratio test (LRT). Sensitivity analyses were performed to include studies with at least 5 individuals in the infected and control groups (rather than 10), and to assess trials in which poliovirus infections were not distinguished from NPEVs. Potential publication bias was assessed using funnel plots and Egger's test [25]. Analyses were conducted in the programming language R [26] and using Review Manager software (RevMan 5.2).

## RESULTS

### Study Selection

**Figure 1.**
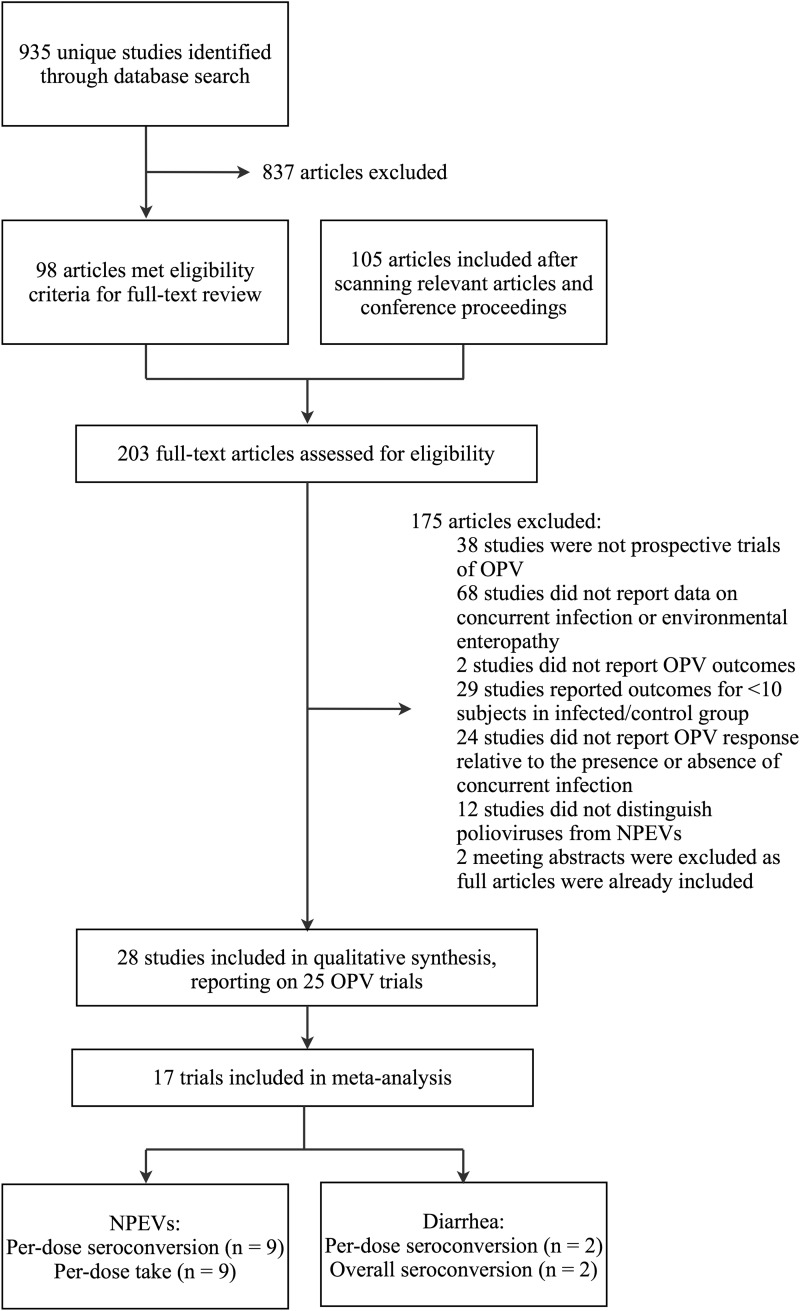
Flow chart of study selection process. A study by Swartz et al [45] was classified as ineligible based on details in a separate report [46], which clarified that the presence of NPEVs infections in the week following (as opposed to preceding) OPV delivery had been used as an indicator of concurrent infection. Citation details of the eligible studies are provided in Table 1. Abbreviations: NPEVs, nonpolio enteroviruses, OPV, oral poliovirus vaccine.

The literature search led to the identification of 203 articles of potential relevance to the review. Among these articles, 28 fulfilled the inclusion criteria, reporting on 25 distinct trials (Table 1) [8–14, 18–20, 27–44]. Figure 1 presents a flow chart of the study selection process.

**Table 1.** Summary of Eligible Trials

Study^a^	Country	Income Group^b^	Vaccine	Doses	Dose Interval	No. OPV Recipients^c^	Age at Recruitment	Vaccination History	Interference Measure						OPV Response^d^			Other Eligible Articles Reporting on Trial
									Diar	NPEV	Vir	Bac	Par	EE	Ser	GMT	Take	
Benyesh-Melnick et al, 1959 [8]	Mexico	UM	mOPV	3^e^	3 wk	81	0–12 y	…	…	✓	…	…	…	…	✓	…	✓	
Fang-Cho, 1960 [27]	China	UM	tOPV	1	…	600	<7 y	…	…	✓	…	…	…	…	✓	…	…	
Levine & Goldblum, 1960 [28]	Israel	H	tOPV	≤2	4 mo	Approximately 500	0–4 mo	…	…	✓	…	…	…	…	…	…	✓	
Voroshilova et al, 1960 [29]	Russia	UM	tOPV	2	4–6 wk	140	2 mo–15 y	…	…	✓	…	…	…	…	…	…	✓	
Domok et al, 1961 [30]	Hungary	H	tOPV	2	6 wk	160	3 mo–15 y	3 or 4 IPV in most	…	✓	…	…	…	…	…	…	✓	
Ramos-Alvarez, 1961 [31]	Mexico	UM	mOPV	3^e^	3 wk	181	<3 y	…	…	✓	…	…	…	…	✓	…	✓	
Dardanoni et al, 1962 [32]	Italy	H	mOPV	1	…	Approximately 55	3 mo–2 y	some IPV	…	✓	…	…	…	…	✓	…	…	
Ingram et al, 1962 [33]	USA	H	mOPV	1	…	25	10 wk–8 mo	some IPV	…	✓	…	…	…	…	✓	…	✓	
Paul et al, 1962 [34]	Costa Rica	UM	tOPV	2	1 mo	48	5–22 mo	some IPV	…	✓	…	…	…	…	✓	…	✓	Paul et al [35]
Urasawa, 1964 [19]	Japan	H	tOPV	1	…	NA	NA	…	…	✓	…	…	…	…	✓	…	…	
Spano et al, 1965 [36]	Italy	H	mOPV	3^e^	1 mo	229	3 mo–5 y	…	…	✓	…	…	…	…	…	…	✓	
JLPRC, 1966 [9]^f^	Japan	H	mOPV	3^e^	4 wk	Approximately 5000	0–60 y	some IPV	…	✓	…	…	…	…	✓	✓	✓	Urasawa [19, 37], Takatsu [38]
Nardi et al, 1966 [39]^g^	Italy	H	tOPV	1	…	197	1–10 y	3×mOPV^e^	…	✓	…	…	…	…	✓	✓	✓	Monaci et al [40]
Ramos-Alvarez, 1966 [18]	Mexico	UM	mOPV	3^e^	4 wk	NA	7 mo–2 y	…	…	✓	…	…	…	…	✓	…	✓	
Ramos-Alvarez, 1966 [18]	Mexico	UM	tOPV	1	…	NA	7 mo–2 y	…	…	✓	…	…	…	…	✓	…	…	
Sureau et al, 1966 [41]	Algeria	UM	tOPV	3	4 wk	100	3 mo–3 y	…	…	✓	…	…	…	…	…	…	✓	
John & Christopher, 1975 [14]	India	LM	tOPV	2	8 wk	191	3 mo–5 y	…	…	✓	…	…	…	…	✓	…	✓	
Mahmoud et al, 1976 [11]	Egypt	LM	NA	1	…	24	3 mo–2 y	…	✓	…	…	✓	…	…	…	…	✓	
Faden et al, 1992 [42]	USA	H	tOPV	≤3	2–8 mo	68	6–10 wk	…	…	✓	✓	…	…	…	✓	✓	…	
Kok et al, 1992 [13]	Kenya	L	tOPV	3	2 mo	100	2–3 mo	…	✓	✓	…	…	…	…	✓	✓	…	
WHO, 1995 [20]	Gambia	L	tOPV	4	NA	1087	<6 wk	…	✓	…	…	…	…	…	✓	…	…	
Myaux et al, 1996 [43]	Bangladesh	L	tOPV	3	4 wk	391	6–16 wk	…	✓	…	✓	…	…	…	✓	✓	…	
Posey et al, 1997 [12]	Brazil	UM	tOPV	4	4–6 wk	1395	0 (birth)	…	✓	…	…	…	…	…	✓	…	…	WHO [20]
Maldonado et al, 1997 [4,4]	Mexico	UM	tOPV	2	8 wk	237	6 wk–6 mo	…	…	✓	✓	✓	…	…	✓	✓	…	
Triki et al, 1997 [10]	Tunisia	UM	tOPV	3	4 wk	121	3 mo	…	…	✓	✓	✓	✓	…	✓	…	…	

Abbreviations: Bac, enteric bacteria; Diar, diarrhea; EE, environmental enteropathy; GMT, geometric mean titer; H, high; IPV, inactivated poliovirus vaccine; JLPRC, Japan Live Poliovaccine Research Commission; L, low; LM, lower middle; mOPV, monovalent oral poliovirus vaccine; NA, not reported; NPEV, nonpolio enterovirus; OPV, oral poliovirus vaccine; Par, eukaryotic parasites; Ser, seroconversion; tOPV, trivalent oral poliovirus vaccine; UM, upper middle; Vir, other viruses (nonenteroviruses); WHO, World Health Organization.

^a^ Where multiple reports describing the same trial fulfilled the eligibility criteria for inclusion in the review, the most comprehensive report is listed here.

^b^ Current income status designated by the World Bank [24].

^c^ The total number of OPV recipients in each trial is indicated; however, the influence of enteric infections was often examined in a subset of the total study population.

^d^ Outcomes are listed if they were reported within the eligible timeframe (ie, within 8 weeks of vaccination for serological response and between 1 and 4 weeks for vaccine take) and compared according to the presence or absence of infection at the time of vaccine delivery; other OPV outcomes may have been reported in each study, but not with respect to enteric infection or within the eligible timeframe.

^e^ Three doses of mOPV were delivered in the order type 1, type 3, type 2.

^f^ The study reported on a collaborative trial of mOPV involving 27 laboratories across Japan, commencing in May 1961; data from preceding eligible reports, using the same vaccine formulation and also commencing in May 1961 (by Urasawa [19, 37] and Takatsu [38]) were assumed to be included in or overlap with this later and more comprehensive report in order to avoid the risk of duplicating data from individual vaccinees. However, the study by Urasawa [19] also reported on a trial of tOPV, which was included in the meta-analysis separately.

^g^ Data regarding vaccine take were obtained from the report by Monaci et al [40], which included a subset of the children under 5 years of age reported on by Nardi et al [39], and an additional 70 vaccinees 6–10 years of age.

### NPEV Coinfection

#### Serological Response

**Figure 2.**
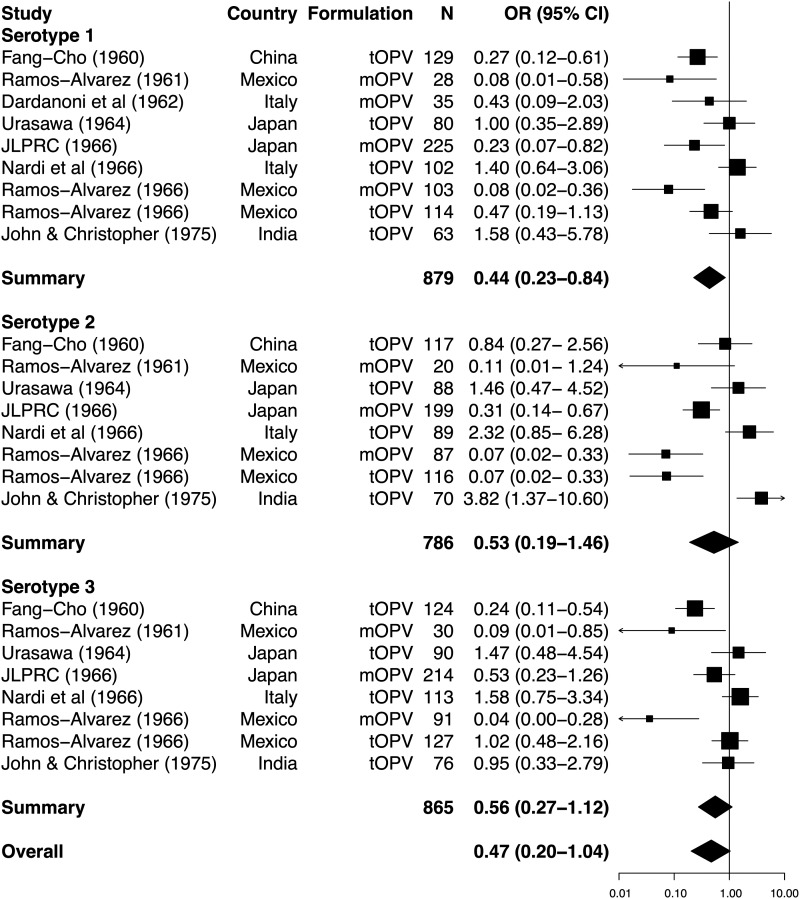
Forest plot of the impact of concurrent nonpolio enterovirus infections on the odds of seroconversion per dose of oral poliovirus vaccine. ORs and 95% CIs, calculated using random-effects models, are presented for each serotype by boxes and black lines, with box area proportional to study weight. Summary ORs for each serotype are indicated by a diamond, the width of which represents the 95% CI. The overall OR was calculated by maximum likelihood estimation using a multilevel meta-analytic model based on structural equation modeling, incorporating study as a cluster effect. Abbreviations: CI, confidence interval; JLPRC, Japan Live Poliovaccine Research Commission; mOPV, monovalent oral poliovirus vaccine; N, number of vaccinees; OR, odds ratio; tOPV, trivalent oral poliovirus vaccine.

**Figure 3.**
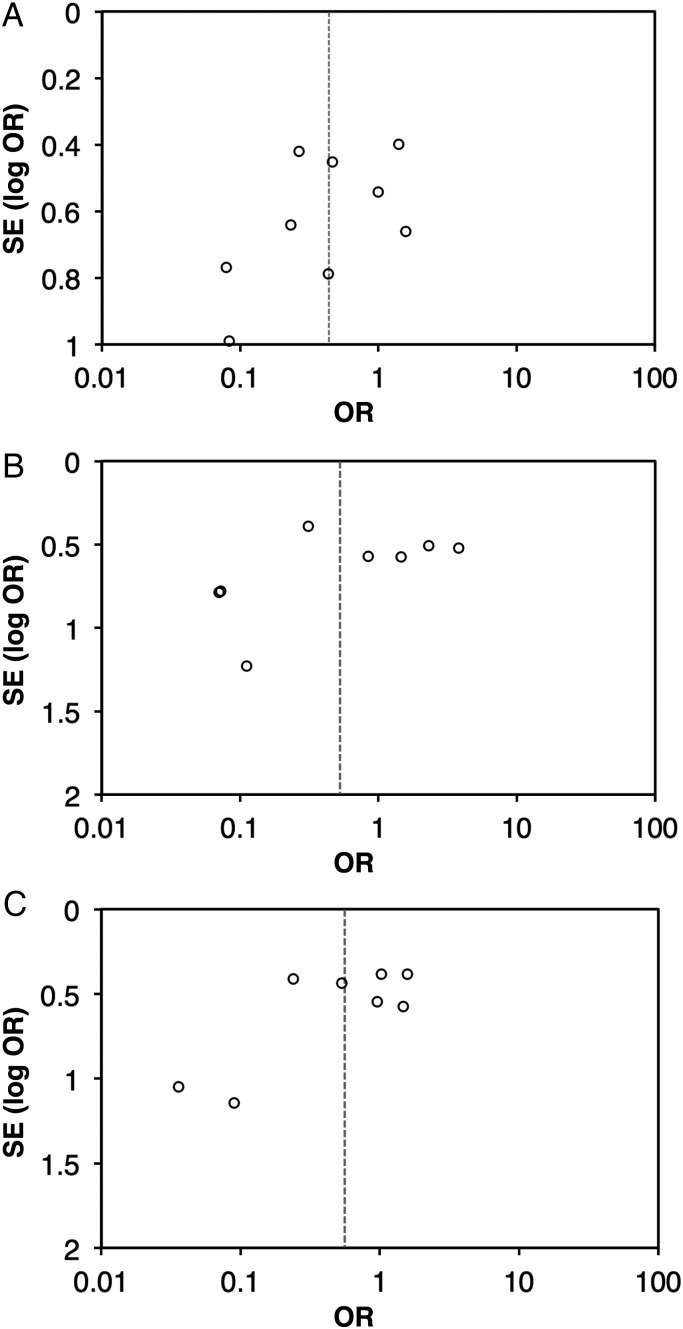
Funnel plots for the influence of nonpolio enterovirus infections on the odds of seroconversion per dose of oral poliovirus vaccine. Data are presented for serotype 1 (*A*), serotype 2 (*B*), and serotype 3 (*C*). Abbreviations: OR, odds ratio; SE, standard error.

Sixteen eligible studies reported serological response to OPV according to the presence or absence of NPEVs (Supplementary Table 1). Across 9 studies eligible for inclusion in the meta-analysis (all involving delivery of Sabin vaccine), the presence of NPEVs had a significant inhibitory effect on seroconversion rates for type 1 poliovirus, but not types 2 or 3 (Figure 2). Overall, the reduction in per-dose seroconversion among NPEV-infected individuals approached significance (summary OR, 0.47; 95% CI, .20−1.04). There was no evidence of marked publication bias (Egger's test, *P* > .1 for each serotype; Figure 3). Serotype did not significantly influence effect size (LRT, *P* = .680). Significant heterogeneity in ORs across studies was observed for each serotype (χ^2^, *P* values .002, <.001, and <.001 for types 1, 2, and 3, respectively) and overall (χ^2^, *P* < .001).

**Figure 4.**
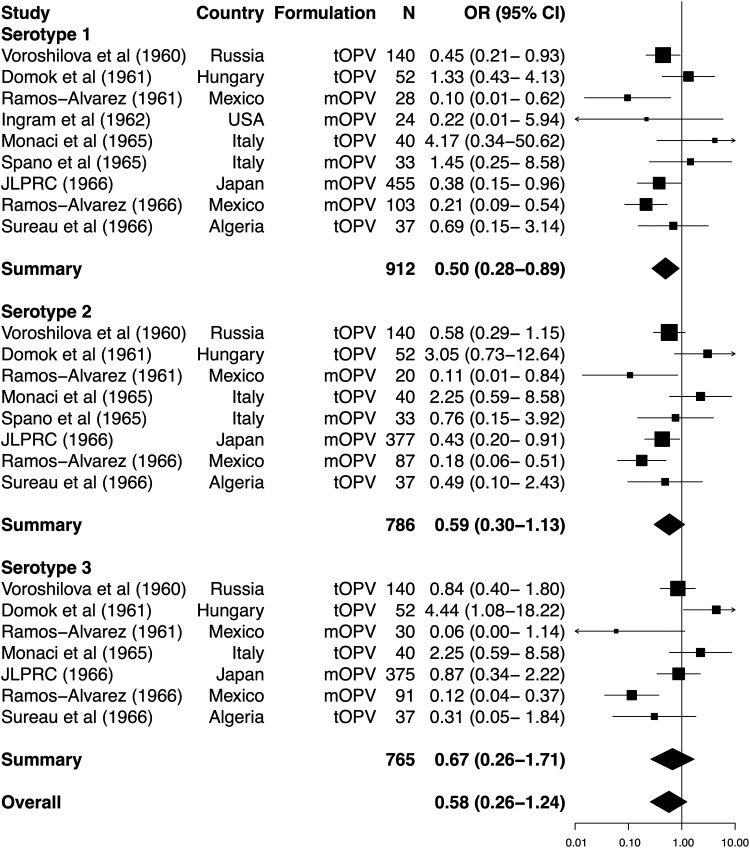
Forest plot of the impact of concurrent nonpolio enterovirus infections on the odds of vaccine shedding per dose of oral poliovirus vaccine. See Figure 2 for labeling. Abbreviations: CI, confidence interval; JLPRC, Japan Live Poliovaccine Research Commission; mOPV, monovalent oral poliovirus vaccine; N, number of vaccinees; OR, odds ratio; tOPV, trivalent oral poliovirus vaccine.

When the meta-analysis was stratified according to OPV formulation, NPEV-associated interference was significant for each serotype following the administration of mOPV (summary OR, 0.17; 95% CI, .07−.42), but not tOPV (summary OR, 0.92; 95% CI, .45−1.86; Supplementary Table 2). The impact of formulation on effect size was significant (LRT, *P* = .008). A separate subgroup analysis revealed that interference was generally greater among studies conducted in low-, lower-middle-, or upper-middle-income than high-income countries (Supplementary Table 2), although the impact of trial setting was not significant (LRT, *P* = .181).

A sensitivity analysis including studies with a minimum of 5 individuals in the infected and control groups did not alter the outcomes of this meta-analysis (Supplementary Table 2). However, when the inclusion criteria were broadened to incorporate studies that did not distinguish polioviruses from NPEVs, the inhibitory effect of concurrent infection was significant overall (summary OR, 0.55; 95% CI, .33−.89; Supplementary Materials Section 2.1).

A limited number of studies examined whether concurrent NPEVs influenced seroconversion after multiple doses of tOPV [13, 14, 34, 44]. The influence of NPEVs on overall seroconversion rates remains unclear based on the small number and heterogeneous nature of these studies (Supplementary Materials Section 2.2).

#### Vaccine Take

The shedding of vaccine poliovirus has frequently been used as a marker of OPV take owing to its strong correlation with serological response. Nine studies reporting vaccine take according to the presence or absence of NPEVs were eligible for inclusion in the meta-analysis (Figure 4). Sabin vaccine was used in each study (where specified). As with serological response, a significant decrease in the odds of shedding in NPEV-infected individuals was observed for serotype 1, but not types 2 or 3. There was no evidence of marked publication bias (Egger's test, *P* > .1 for each serotype). Overall, the reduction in shedding among NPEV-infected individuals was not significant (summary OR, 0.58; 95% CI, .26−1.24). The impact of serotype was not significant following meta-regression (LRT, *P* = .489). Heterogeneity among studies was significant for serotypes 2 and 3 (χ^2^, *P* values .081, .015, and .001 for types 1, 2, and 3, respectively) and for the overall OR (χ^2^, *P* < .001). Again, stratification of the meta-analysis according to vaccine formulation revealed that NPEV-associated interference was greater for mOPV than tOPV (LRT, *P* = .032), and in low-, lower-middle-, or upper-middle-income than high-income countries (LRT, *P* = .019; Supplementary Table 2).

#### Impact of Specific NPEVs

In 7 studies, the influence of NPEVs on OPV take and/or seroconversion was reported according the presence of specific pathogens [19, 28, 32, 33, 36] or pathogen groups [14, 27]. The numbers of individuals infected with particular NPEVs were generally too small to enable the influence of specific pathogens to be evaluated, and there was no strong evidence to support the particular inhibitory effect of any specific NPEV or NPEV group (Supplementary Materials Section 2.3).

### Concurrent Diarrhea

#### Serological Response

**Figure 5.**
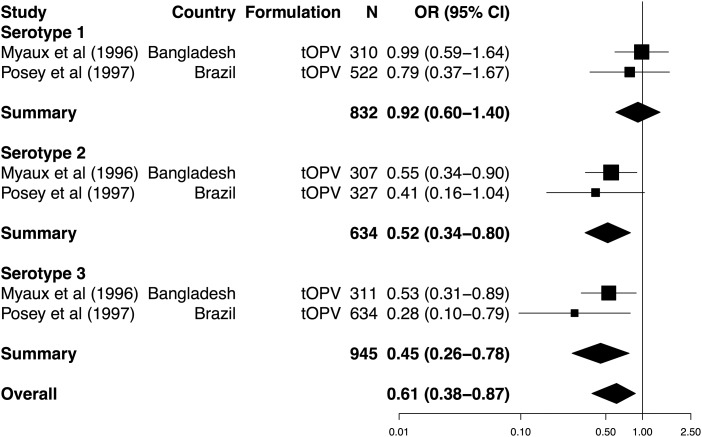
Forest plot of the impact of concurrent diarrhea on the odds of seroconversion per dose of oral poliovirus vaccine. See Figure 2 for labeling. Abbreviations: CI, confidence interval; N, number of vaccinees; OR, odds ratio; tOPV, trivalent oral poliovirus vaccine.

Four of the included trials considered the influence of concurrent diarrhea on serological response to tOPV (Supplementary Table 3), of which 2 were eligible for inclusion in the meta-analysis [12, 43]. Both studies involved the delivery of Sabin vaccine. Concurrent diarrhea was associated with a significant decrease in per-dose seroconversion for serotypes 2 and 3, but not type 1 (Figure 5). Overall, the impact of diarrhea on seroconversion was significant (summary OR, 0.61; 95% CI, .38−.87). The impact of serotype on effect size approached significance (LRT, *P* = .067). Heterogeneity among studies was not significant for any serotype (χ^2^, *P* values > .1) or overall (χ^2^, *P* = .207).

**Figure 6.**
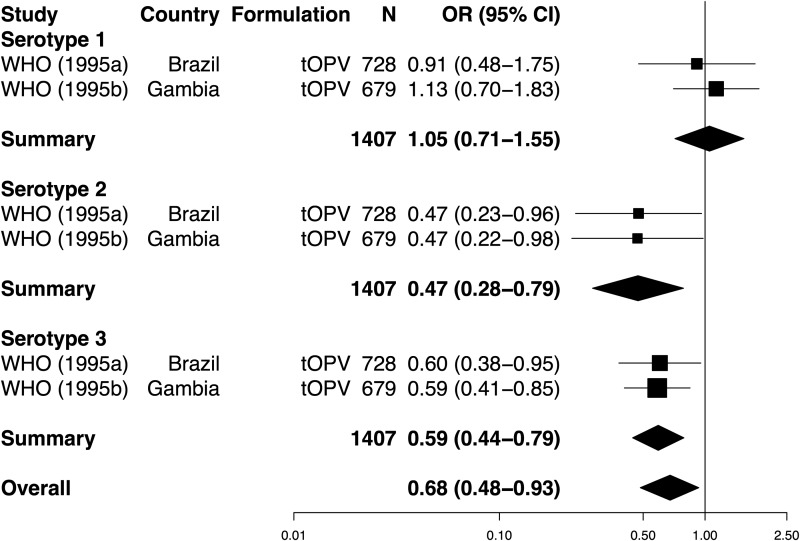
Forest plot of the impact of concurrent diarrhea on the odds of seroconversion following multiple doses of oral poliovirus vaccine. See Figure 2 for labeling. Data were obtained from 1 article [20], reporting on separate trials of tOPV in Brazil ([WHO] 1995a) and the Gambia ([WHO] 1995b). Abbreviations: CI, confidence interval; N, number of vaccinees; OR, odds ratio; tOPV, trivalent oral poliovirus vaccine; WHO, World Health Organization.

We carried out a separate analysis to compare seroconversion rates after multiple tOPV doses among individuals experiencing diarrhea at the time of 1 or more doses with those free of diarrhea at every dose (Figure 6). Overall, a significant decline in the odds of seroconversion was observed in children who experienced at least 1 concurrent diarrheal episode (summary OR, 0.68; 95% CI, .48−.93). This interference effect was significant for serotypes 2 and 3, but not type 1. Heterogeneity among studies was not significant for any serotype (χ^2^, *P* values > .1) or overall (χ^2^, *P* = .154). Effect size differed significantly according to serotype (LRT, *P* = .024). Notably, 2 other studies did not observe a significant impact of concurrent diarrhea on serological response after multiple doses of tOPV, but were not eligible for inclusion in this analysis [13, 43]. There were insufficient studies to formally assess publication bias regarding diarrhea-associated interference.

#### Vaccine Take

One trial considered the influence of concurrent diarrhea on OPV take [11]. Over a 3-week period after OPV delivery, vaccine shedding was observed in 10% (1/10) of infants with diarrhea at the time of immunization, and 64.3% (9/14) of those without diarrhea.

### Other Enteropathogens

Five studies considered the effect of enteric infections other than NPEVs on OPV outcomes [10, 11, 42–44]. Although these reports provide some indication of a potential inhibitory effect of enteric bacteria [11, 44] and protozoa [10] on response to OPV (Supplementary Materials Section 2.4), the extent of this interference remains uncertain owing to the small number and heterogeneous nature of the available studies.

### Environmental Enteropathy

No studies encountered in this review examined the influence of environmental enteropathy on OPV response.

## DISCUSSION

The impaired immunogenicity of OPV in low-income countries has been consistently documented since the early field trials of this vaccine. However, explanations for this phenomenon remain poorly resolved. Our systematic review supports the role of enteric infections as a risk factor for impaired OPV response: for type 1 poliovirus, a significant reduction in the odds of seroconversion and vaccine take was observed in NPEV-infected individuals, while concurrent diarrhea significantly inhibited seroconversion for serotypes 2 and 3. Enteropathogens other than NPEVs may also interfere with response to OPV, but have been considered by only a small number of studies to date.

Significant heterogeneity in the extent of NPEV-associated interference was observed among studies included in this review. Factors that may contribute to this heterogeneity include vaccine potency, age at vaccination, collection method for fecal samples, viral isolation methods, and the starting dilutions and seroconversion thresholds adopted during neutralization tests, which varied widely among studies (Supplementary Table 1). Stratification of the analysis revealed that interference with both take and seroconversion was generally greater following the delivery of mOPV than tOPV, and in trials conducted in low- or middle-income as opposed to high-income countries. The trends were consistent across serotypes (Supplementary Table 2). These stratified findings should be interpreted with caution given the small number of studies in each subgroup, and the potential for individual studies with marked interference effects (eg, Ramos-Alvarez [18, 31]) to exert a strong influence on a particular subgroup. Nonetheless, they raise the possibility that mOPV may be more susceptible than tOPV to the influence of enteric infections. This observation may reflect the dynamics of competition between enteroviruses coinciding in the gut: while an established NPEV infection may have a marked inhibitory effect on a single attenuated vaccine strain in mOPV, the additive effect on top of the interserotype interference arising between poliovirus strains in tOPV may be smaller. The disparity in effect according to trial setting suggests that individuals in low- or middle-income countries may be more susceptible to NPEV-associated interference. However, one must also consider the possibility that individuals in these regions who are—for other reasons (eg, malnutrition)—at risk of OPV nonresponse may be more susceptible to NPEV infections, and that the apparent association between NPEVs and impaired OPV response is not causal.

At present, we can only speculate as to the potential mechanisms that may account for the observed interference effects. Although NPEVs do not use the poliovirus receptor (CD155) to access cells, their binding to nearby receptors may impede the attachment and entry of vaccine polioviruses. The potential for species C enteroviruses to infect the same cells as polioviruses is supported by the observation of recombinant strains in circulating vaccine-derived polioviruses [47]. Interference by NPEVs or other enteropathogens may also arise indirectly via the induction of nonspecific innate antiviral immunity, or—in the case of diarrhea—by reducing the gut's mucosal surface area (and hence access to poliovirus receptors) and increasing the rate of gastrointestinal transit.

The inhibitory effect of NPEVs was more pronounced for serotype 1 than types 2 or 3, while the opposite was true of diarrhea-associated interference. Although the differences were generally not significant following meta-regression, these findings suggest that the extent of interference may differ among serotypes, and that this specificity may vary according to the nature of the concurrent infection. The notion that Sabin type 1 poliovirus may be more susceptible to interference by concurrent enteroviruses is consistent with the need for an elevated potency of this serotype within the tOPV formulations currently in use, while the enhanced replicative fitness of Sabin type 2 virus may be associated with a greater resistance to these interference effects [2].

The relative influence of individual NPEVs or NPEV groups on OPV response remains unclear based on the available evidence. While Urasawa [19, 37] highlighted the potential inhibition of mOPV responses by Coxsackie B5 virus, this pathogen did not interfere with seroconversion following tOPV delivery. Moreover, studies carried out in India [14] and China [27] documented comparable seroconversion rates in individuals infected with Coxsackie A, Coxsackie B, and ECHO/other viruses at tOPV delivery, albeit with small numbers of individuals in each group.

Several limitations of the present review should be acknowledged. By considering the influence of enteric infections 0–7 days preceding vaccination, the study did not account for the possible effects of the stage of infection on the outcomes of coinfection [48], or the potential influence of infections arising after OPV delivery [29, 45, 46]. In addition, a history of exposure to enteropathogens may induce inflammatory and other changes in the innate immune status of the intestinal mucosa that affect OPV response even in the absence of concurrent infection, or give rise to a state of mucosal or systemic immune tolerance that diminishes OPV response. Environmental enteropathy has been linked with exposure to enteropathogens, and may contribute to the impaired immune response to OPV in low-income settings [16]. However, we encountered no studies reporting on the association between markers of this subclinical disorder and OPV response.

Another important consideration is the general tendency of the included studies to focus on a specific group of enteropathogens. Only 2 studies examined the influence of both viral and nonviral pathogens on OPV responses [10, 44], while the use of only monkey kidney cells for enterovirus detection by several trials [8, 9, 28, 30, 33] would result in certain NPEV infections going undetected [49]. Given the pervasive nature of enteropathogens [50], it is likely that many individuals classified as “controls” during the present analysis harbored other pathogens at the time of vaccination. This could result in an underestimate of the association between NPEVs and vaccine nonresponse. Conversely, coinfection with other pathogens among individuals harboring NPEVs may be common given the shared risk factors for infection. An effect of these coinfections may be captured by the meta-analysis, which would bias our findings in the opposite direction.

Of the 25 studies included in the present review, it is notable that none were performed in the last 15 years. This may relate to the costly, labor-intensive efforts required to detect enteropathogens, as well as a gradual shift away from the early conviction in the importance of concurrent infections as an inhibitor of OPV response [2, 14]. Given recent advances in molecular diagnostics, pathogen detection is no longer the cumbersome task it was when OPV was first introduced. There is considerable potential, therefore, to return to the question of whether enteropathogens, either through concurrent infection or enteropathy, contribute to the impaired immunogenicity of OPV in low-income countries. Further research is warranted to determine the relative influence of particular pathogens, as well as the potential role of the gut microbiota as a whole in shaping OPV response. These questions are relevant not only to polio eradication efforts, but to the use of oral vaccines in general. While the present review validates the potential contribution of enteric infections to impaired oral vaccine performance in low-income settings, further research is required to determine the full extent of this contribution, as well as the best strategies toward overcoming its detrimental effects.

## Supplementary Data

Supplementary materials are available at *The Journal of Infectious Diseases* online (http://jid.oxfordjournals.org/). Supplementary materials consist of data provided by the author that are published to benefit the reader. The posted materials are not copyedited. The contents of all supplementary data are the sole responsibility of the authors. Questions or messages regarding errors should be addressed to the author.

Supplementary Data
