# Supplementary material for: Influence of Enteric Infections on Response to Oral Poliovirus Vaccine: A Systematic Review and Meta-analysis
Source: J Infect Dis. 2014 Mar 31;210(6):853–64. doi: 10.1093/infdis/jiu182 (PMC4136801; doi:10.1093/infdis/jiu182)
Supplement: Supplementary Data [file supp_210_6_853__index.html]

Influence of Enteric Infections on Response to Oral Poliovirus Vaccine: A Systematic Review and Meta-analysis — Supplementary Data 

# Influence of Enteric Infections on Response to Oral Poliovirus Vaccine: A Systematic Review and Meta-analysis

## Supplementary Data

Supplementary Data

**Files in this Data Supplement:**

- Supplementary Data - Pdf file
- Supplementary Tables - pdf file
