# Supplementary material for: Influence of Enteric Infections on Response to Oral Poliovirus Vaccine: A Systematic Review and Meta-analysis
Source: J Infect Dis. 2014 Mar 31;210(6):853–64. doi: 10.1093/infdis/jiu182 (PMC4136801; doi:10.1093/infdis/jiu182)
Supplement: Supplementary Data [file supp_jiu182_jiu182supp_data.pdf]

SUPPLEMENTARY MATERIALS

1. Supplementary Methods

1.1. Search Term

Titles, key words, and abstracts in the citation databases PubMed and ISI Web of Knowledge were searched using the following term: "polio* and (oral or OPV) and (compet* or interfer* or diarrh* or entero* or enteric or coinfect* or bacter* or helminth* or parasit* or protozoa*) and (immunogen* or stool or excret* or shed* or sero* or serum or antibod* or efficac* or fec* or faec*)". The asterisk symbols in this search term function as wildcards enabling the matching of truncated words.

1.2. Screening of Abstracts and Titles

Following the identification of potentially relevant studies based on the search of PubMed and ISI Web of Knowledge, articles were considered eligible for full-text review if their abstracts reported the delivery of oral poliovirus vaccine (OPV) to human recipients alongside the assessment of: (1) concurrent diarrhea; (2) enterovirus excretion or fecal sample collection at or near the time of vaccination; (3) other indicators of concurrent enteric infection; (4) environmental enteropathy; or (5) unspecified factors influencing or interfering with OPV response. Articles were excluded if they did not report on primary research or included less than 10 OPV recipients. Among the articles for which no abstracts were available, full-text copies were obtained if the titles referred to OPV delivery alongside 1 of the 5 potential indicators of interference listed above. Eligible articles were obtained from electronic journals or from the holdings of the British Library (London, UK) or Wellcome Library (London, UK).

1.3. Search for Additional Studies

Additional studies were identified by scanning the text and bibliographies of relevant articles − including any article discussing the phenomenon of interference by enteric infections on OPV response − being considered for full-text review. Articles were obtained if they were noted to have examined the effect of nonpolio enteric infections, diarrhea, or environmental enteropathy on OPV response, or if their title referred to OPV and 1 of these potential indicators of enteric interference. Several complete conference proceedings [1-3] were also scanned, as were a number of relevant articles [4-11] identified during the review of abstracts or known to the authors.

2. Supplementary Results

2.1. Enterovirus Coinfection

Studies were not included in the primary analysis if the reporting of enterovirus coinfection did not distinguish concurrent poliovirus infections (wild-type or vaccine-derived) from nonpolio enteroviruses (NPEVs). This specification was included to avoid the risk of biasing the results in favor of an interference effect, given the known interserotype interference that arises between the vaccine poliovirus strains [6]. The inability of OPV to replicate in the face of existing poliovirus infections is also supported by the failure of additional doses of heterotypic monovalent OPV (mOPV) to interrupt poliovirus excretion in individuals with immunodeficiency-related vaccine-derived poliovirus infections (although this has been tested in only a small number of cases to date) [12]. However, we performed a sensitivity analysis to incorporate studies in which the reporting of data did not enable poliovirus-infected individuals to be excluded.

Following the inclusion of 6 additional studies [13-18], significant inhibition of per-dose seroconversion was evident for type 1 poliovirus (odds ratio [OR] 0.51, 95% confidence interval [CI] .34−.75), but not for type 2 or 3 virus (OR 0.67, 95% CI .33−1.36 and OR 0.58, 95% CI .33−1.02, respectively). Inhibition of per-dose seroconversion was significant overall (summary OR 0.55, 95% CI .33−.89; Supplementary Table 2). The additional studies involved the delivery of Sabin [13, 15], non-Sabin [16], and unspecified [14, 17] OPV strains. Heterogeneity, calculated using the χ^2 statistic, was significant for each poliovirus serotype (*P* values .012, <.001, and <.001 for type 1, 2, and 3 data, respectively) and for the overall OR ($P < .001$). During meta-regression, effect size did not

differ significantly according to poliovirus serotype (likelihood ratio test [LRT], P = .434), and in contrast to the primary analysis, was not significantly affected by formulation (mono-, bi-, or tri-valent OPV; LRT, P = .248) or trial setting (LRT, P = .303). Inclusion of 1 extra study involving delivery of non-Sabin OPV in the analysis of vaccine take did not markedly change the results (Supplementary Table 2) [16]. Since the primary focus of this review was to assess the impact of nonpolio enteric infections on OPV response, poliovirus-infected individuals were still excluded from this sensitivity analysis where possible. The proportion of enteroviruses accounted for by polioviruses, where reported, varied from less than 10% [15] to more than 50% [18].

Several studies also examined the influence of enterovirus infections (not excluding polioviruses) at the time of the first OPV dose on overall seroconversion rates after multiple doses. Pangi et al [19] observed a significant decrease in seroconversion rates for type 1 and 3 virus after 3 doses of trivalent OPV (tOPV) among individuals harboring enteroviruses during delivery of the first dose. However, a significant inhibitory effect was not observed in several other studies involving between 3 and 5 doses of tOPV [20-22]. Again, polioviruses accounted for between 10% and more than 50% of the enteroviruses isolated from prevaccination samples in these studies.

2.2. Impact of Concurrent NPEV Infections Across Multiple Doses of tOPV

A limited number of trials examined the influence of NPEV infections at the time of 1 or more doses on seroconversion rates after multiple doses of tOPV. Maldonado et al [23] reported a significant decrease in type 1 seroconversion rates after 2 doses of tOPV in infants presenting with concurrent NPEV infection (including any infection identified in stool samples 1 week before, at the time of, or 1 week after OPV delivery) compared with those free of NPEV infections. By contrast, Kok et al [24] reported no significant differences in the antibody levels obtained after 3 doses of tOPV relative to the presence or absence of NPEV infections at the time of vaccine delivery, while John and Christopher [25] did not observe significant inhibition of vaccine take or seroconversion among NPEV-infected individuals seronegative to 1 or more poliovirus serotypes upon receipt of a second dose of tOPV. Heterogeneity in the reporting of these trials precluded the synthesis of data across studies.

2.3. Impact of Specific NPEVs

Seven studies reported the influence of NPEVs on OPV take and/or seroconversion rates according the presence of specific pathogens [26-30] or pathogen groups [25, 31]. Although the numbers of individuals affected by particular NPEVs were generally small, the findings of 4 studies merit particular mention. Ingram et al [28] documented vaccine response following the delivery of type 1 mOPV during an epidemic of ECHO 14 virus in an infant population. The virus did not appear to inhibit seroconversion rates or vaccine take, although the study included a total of only 25 vaccinees. Urasawa [29] reported an inhibitory effect of Coxsackie B5 virus on both take and serological response following the delivery of mOPV; however, Coxsackie B5 virus did not inhibit serological response to tOPV (Figure 2). In a study by Fang-Cho [31] involving 1 dose of tOPV, interference with seroconversion was similar for different subgroups of NPEVs (Coxsackie A, Coxsackie B, or ECHO/ other virus). On the other hand, John and Christopher [25] observed no interference with seroconversion irrespective of the subgroup of NPEV present at the time of tOPV administration.

2.4. Other Enteric Pathogens

We identified only 5 studies that reported the effect of enteric infections other than NPEVs on response to OPV [23, 32-35]. Although several other studies used isolation techniques permissive to the detection of adenoviruses or reoviruses (eg, [25, 30]), either the infections were not observed in prevaccination samples or their impact on OPV response was not reported.

Myaux et al [32] examined rectal swabs for rotavirus antigen before delivery of the first of 3 tOPV doses. Among 11 individuals positive for rotavirus antigen with complete follow-up data, low seroconversion rates were observed for all 3 serotypes following 1 dose of OPV compared with individuals negative for rotavirus antigen (although the differences were not significant). Geometric mean titers (GMTs) were also diminished in rotavirus-positive compared with -negative individuals after 1 dose, but not after 3 doses.

Maldonado et al [23] examined the combined influence of concurrent viral pathogens (including NPEV, adenovirus 40/41, astrovirus, and rotavirus infections) and enteric bacteria on seroconversion rates across 2 doses of tOPV: concurrent infections were generally more frequent among

nonresponders compared with responders at each dose, and were linked with a diminished response to type 1 and 2 poliovirus at the second dose. GMTs for type 1 and 2 poliovirus were significantly lower among infants with concurrent viral or bacterial infections compared with those lacking infections after the first dose of OPV, and for type 2 poliovirus after the second dose. The authors also noted a significant decrease in serological response to type 2 poliovirus after 2 doses among infants infected with astrovirus and enteric bacterial infections compared with uninfected individuals. Notably, in spite of the significant interference effects for type 1 and 2 poliovirus observed in this study, overall seroconversion rates for these serotypes after 2 doses of tOPV were 86% and 97%, respectively. Corresponding seroconversion rates for type 3 poliovirus were only 61% after 2 doses, though no significant interference effects were observed for this serotype.

Triki et al [33] measured the influence of enteric bacteria and eukaryotic parasites on serological response to tOPV. Overall seroconversion rates were high in this study, with only 16 out of 114 individuals failing to seroconvert to all 3 poliovirus serotypes after 3 doses. Among individuals with incomplete seroconversion, a higher frequency of enteric parasites − including *Candida albicans*, *Candida tropicalis*, and *Giardia intestinalis* − was observed (5/16 [31%], compared with 12/98 [12%] in those with complete seroconversion). The prevalence of enteric bacteria, rotaviruses, and adenoviruses did not differ significantly between these groups. It should be noted, however, that stool samples were obtained both at the time of vaccination and during intervening episodes of diarrhea in this study; the reporting of enteric pathogen prevalence therefore included − but was not limited to − infections coinciding with OPV delivery.

In a study examining the influence of concurrent enteric and nasopharyngeal viral pathogens (including NPEV, adenovirus, and rotavirus infections) on response to OPV and/or enhanced-potency inactivated poliovirus vaccine (IPV), Faden et al [34] reported detectable antibodies to each poliovirus serotype in all participants after 3 vaccine doses, irrespective of immunization schedule (OPV alone, IPV alone, or combined). Compared with uninfected controls, individuals with viral infections had similar serum neutralizing antibody, nasopharyngeal neutralizing antibody, and nasopharyngeal immunoglobulin A GMTs following the delivery of tOPV doses at either 4 or 12 months of age, though a modest decrease in serum antibody titers was evident for the latter dose among infected individuals. Interference was examined in a small number of tOPV recipients in this study.

Finally, in a trial by Mahmoud et al [35], in which concurrent diarrhea was observed to reduce OPV take, albeit in a total of 24 vaccinees, each case of diarrhea was attributed to *Shigella* infection.

References

1. Proceedings of the First International Conference on Live Poliovirus Vaccines, Scientific Publication No. 44. Pan American Sanitary Bureau, **1959**.
2. Proceedings of the Second International Conference on Live Poliovirus Vaccines. Scientific Publication No. 50. Pan American Sanitary Bureau, **1960**.
3. Oral Live Poliovirus Vaccine: Papers presented at the IVth Scientific Conference of the Institute of Poliomyelitis and Virus Encephalitis and the International Symposium on Live Poliovirus Vaccine, **1960**.
4. Salk J. Immunization against poliomyelitis: risk/benefit/cost in a changing context. Dev Biol Stand **1979**; 43:151–7.
5. Sabin AB. Vaccination against poliomyelitis in economically underdeveloped countries. Bull World Health Organ **1980**; 58:141–57.
6. Patriarca PA, Wright PF, John TJ. Factors affecting the immunogenicity of oral poliovirus vaccine in developing countries: review. Rev Infect Dis **1991**; 13:926–39.
7. Tulchinsky T, Abed Y, Shaheen S, et al. A ten-year experience in control of poliomyelitis through a combination of live and killed vaccines in two developing areas. Am J Public Health **1989**; 79:1648–52.
8. Oduntan SO, Lucas AO, Wennen EM. The immunological response of Nigerian infants to attenuated and inactivated poliovaccines. Ann Trop Med Parasitol **1978**; 72:111–5.
9. Grassly NC, Wenger J, Durrani S, et al. Protective efficacy of a monovalent oral type 1 poliovirus vaccine: a case-control study. Lancet **2007**; 369:1356–62.
10. Gerichter CB, Lasch EE, Sever I, el-Massri M, Skalska P. Paralytic poliomyelitis in the Gaza Strip and West Bank during recent years. Dev Biol Stand **1978**; 41:173–7.
11. Fine PE, Carneiro IA. Transmissibility and persistence of oral polio vaccine viruses: implications for the global poliomyelitis eradication initiative. Am J Epidemiol **1999**; 150:1001–21.
12. MacCallum FO. Hypogammaglobulinaemia in the United Kingdom. VII. The role of humoral antibodies in protection against and recovery from bacterial and virus infections in hypogammaglobulinaemia. Spec Rep Ser Med Res Counc (G B) **1971**; 310:72–85.

13. Domok I, Balayan MS, Fayinka OA, et al. Factors affecting the efficacy of live poliovirus vaccine in warm climates. Efficacy of type 1 Sabin vaccine administered together with antihuman gamma-globulin horse serum to breast-fed and artificially fed infants in Uganda. Bull World Health Organ **1974**; 51:333–47.
14. Domok I, Balayan MS. Problems connected with the effectiveness of live poliovirus vaccine in warm climates. In: 10th International Biological Symposium: Proceedings of the Symposium on Stability and Effectiveness of Measles, Poliomyelitis and Pertussis Vaccines, **1976**:45–58.
15. Poliomyelitis Commission of the Western Region Ministry of Health, Nigeria. Poliomyelitis vaccination in Ibadan, Nigeria, during 1964 with oral vaccine (Sabin strains). Bull World Health Organ **1966**; 34:865–76.
16. Paul JR, Horstmann DM, Riordan JT, et al. An oral poliovirus vaccine trial in Costa Rica. Bull World Health Organ **1962**; 26:311–29.
17. Peradze T, Montefiore D, Coker G. Oral poliovirus vaccination and breast feeding. West Afr Med J Niger Pract **1968**; 17:122–4.
18. Campillo Sainz C, de Mucha Macias J, Lopez Pintado F, Zamudio Banderas R, Sanchez Spindola I. Studies on the trivalent antipoliomyelitic vaccine administered by oral route in 1 or 2 doses to children in Mexico. Bol Oficina Sanit Panam **1962**; 52:399–408.
19. Pangi NS, Master JM, Dave KH. Efficacy of oral polio vaccine in infancy. Indian Pediatr **1977**; 14:523–8.
20. Ehrengut W, Agrhaly A, Koch I, et al. Serum conversion rate following oral immunization against poliomyelitis in Mali, Africa 1982. Monatsschr Kinderheilkd **1984**; 132:29–31.
21. Chopra K, Kundu S, Chowdhury DS. Antibody response of infants in tropics to five doses of oral polio vaccine. J Trop Pediatr **1989**; 35:19–23.
22. Idris MZ, Mathur A, Sharma P, Chaturvedi UC, Sharma NL. Oral polio vaccination and factors affecting its efficacy. Indian J Med Res **1980**; 71:671–8.
23. Maldonado YA, Pena-Cruz V, de la Luz Sanchez M, et al. Host and viral factors affecting the decreased immunogenicity of Sabin type 3 vaccine after administration of trivalent oral polio vaccine to rural Mayan children. J Infect Dis **1997**; 175:545–53.
24. Kok PW, Leeuwenburg J, Tukei P, et al. Serological and virological assessment of oral and inactivated poliovirus vaccines in a rural population in Kenya. Bull World Health Organ **1992**; 70:93–103.
25. John TJ, Christopher S. Oral polio vaccination of children in the tropics. III. Intercurrent enterovirus infections, vaccine virus take and antibody response. Am J Epidemiol **1975**; 102:422–8.
26. Levine S, Goldblum N. Virological findings and antibody response of infants fed multiple type oral poliomyelitis vaccine. A preliminary report. In: Proceedings of the Second International Conference on Live Poliovirus Vaccines, Scientific Publication No. 50. Pan American Sanitary Bureau, **1960**:270–6.
27. Dardanoni L, Brancato P, Gullo A. Recent research on the take of attenuated poliomyelitis virus (type I, Sabin LSc strain) in children living in a community with a high level of enteroviral infections. Riv Ist Sieroter Ital **1962**; 37:428–43.
28. Ingram VG, Lepow ML, Warren RJ, Robbins FC. Behavior of Sabin type 1 attenuated poliovirus in an infant population infected with ECHO 14 virus. Pediatrics **1962**; 29:174–80.
29. Urasawa S. Studies on the interference between group B coxsackie viruses and polioviruses. 2. Comparisons of the effects of coxsackie B 5 virus on the immunizations with mono- and polyvalent live poliovirus vaccines. Sapporo Igaku Zasshi **1964**; 26:8–14.
30. Spano C, Dardanoni L. Studies on circulation of enterovirus in samples of the population subjected to oral antipoliomyelitis vaccination. Riv Ist Sieroter Ital **1965**; 40:169–78.
31. Fang-Cho K. Serologic response in children of 0 to 7 years of age to oral administration of Sabin live poliomyelitis vaccine. In: Oral Live Poliovirus Vaccine: Papers presented at the IVth Scientific Conference of the Institute of Poliomyelitis and Virus Encephalitis and the International Symposium on Live Poliovirus Vaccine, **1960**:261–6.
32. Myaux JA, Unicomb L, Besser RE, et al. Effect of diarrhea on the humoral response to oral polio vaccination. Pediatr Infect Dis J **1996**; 15:204–9.
33. Triki H, Abdallah MV, Ben Aissa R, et al. Influence of host related factors on the antibody response to trivalent oral polio vaccine in Tunisian infants. Vaccine **1997**; 15:1123–9.
34. Faden H, Duffy L. Effect of concurrent viral infection on systemic and local antibody responses to live attenuated and enhanced-potency inactivated poliovirus vaccines. Am J Dis Child **1992**; 146:1320–3.
35. Mahmoud AA, Imam IZ, Abozid SS, El Mished AM, Mitkis FA. The influence of bacillary dysentery on the efficiency of oral poliovaccine in Egypt. Gaz Egypt Paediatr Assoc **1976**; 24:82–91.
