# Supplementary material for: Influence of Enteric Infections on Response to Oral Poliovirus Vaccine: A Systematic Review and Meta-analysis
Source: J Infect Dis. 2014 Mar 31;210(6):853–64. doi: 10.1093/infdis/jiu182 (PMC4136801; doi:10.1093/infdis/jiu182)
Supplement: Supplementary Data [file supp_jiu182_jiu182supp_table.pdf]

**Supplementary Table 1. Summary of Studies Examining the Influence of Nonpolio Enteric Viruses on Serological Response and/or Take Following Oral Poliovirus Vaccine Delivery**

| | Enteric Virus Measurement | | | | | Take Measurement | | | Seroconversion Measurement | | | | | | | Take Data | | | | Seroconversion Data | | | |
|---|---|---|---|---|---|---|---|---|---|---|---|---|---|---|---|---|---|---|---|---|---|---|---|
| Study[a] | Sample[b] | Timing, Days Before Vaccine[c] | Cell Lines Tested for Cytopathic Effect | Suckling Mice | Other | Sample | Timing | Multiple[d] | Test | Lowest Dilution Tested | Seroconversion Criteria[e] | Timing | Type | Strain | Dose (log10 TCID50) | IR | IT | UR | UT | IR | IT | UR | UT |
| Benyesh-Melnick et al, 1959 | swab | 0 | MK | NA | NA | swab | 7, 14, 21 d | NA | N | 1:10 | NA (antibody rise) | 3 wk | NA | Sabin | NA | 13 | 47 | 18 | 28 | 14 | 47 | 22 | 28 |
| Fang-Cho, 1960 | stool | 0 | NA | NA | NA | - | - | - | N | 1:4 | 4-fold rise | 1 mo | 1 | Sabin | 5–5.5 | - | - | - | - | 35 | 60[f] | 58 | 69 |
| | | | | | | | | | | | | | 2 | Sabin | 5–5.5 | - | - | - | - | 47 | 54[f] | 56 | 63 |
| | | | | | | | | | | | | | 3 | Sabin | 5–5.5 | - | - | - | - | 30 | 58[f] | 54 | 66 |
| Levine & Goldblum, 1960[g] | swab | 0 | MK | NA | NA | swab | 7, 14, 21, 28 d | yes | - | - | - | - | 1 | NA | 5.7–6.2[h] | 9 | 14 | 52 | 65 | - | - | - | - |
| | | | | | | | | | | | | | 2 | NA | 4.2–5.7[h] | 0 | 14 | 0 | 65 | - | - | - | - |
| | | | | | | | | | | | | | 3 | NA | 5.7–6.2[h] | 9 | 14 | 55 | 65 | - | - | - | - |
| Voroshilova et al, 1960[i] | stool | 0 | MK, HeLa, KB, Detroit-6 | yes | yes[j] | stool | 2x per wk | NA | - | - | - | - | 1 | Sabin | 5 | 15 | 58 | 36 | 82 | - | - | - | - |
| | | | | | | | | | | | | | 2 | Sabin | 5 | 22 | 58 | 42 | 82 | - | - | - | - |
| | | | | | | | | | | | | | 3 | Sabin | 5 | 15 | 58 | 24 | 82 | - | - | - | - |
| Domok et al, 1961[k] | stool | 1–5 (3x) | MK | yes | NA | stool | 3, 5, 12, 15, 22, 24 d | yes | - | - | - | - | 1 | Sabin | 5 | 10 | 19 | 15 | 33 | - | - | - | - |
| | | | | | | | | | | | | | 2 | Sabin | 5 | 16 | 19 | 21 | 33 | - | - | - | - |
| | | | | | | | | | | | | | 3 | Sabin | 5 | 16 | 19 | 18 | 33 | - | - | - | - |
| Ramos-Alvarez, 1961 | swab | 0 | HK | NA | NA | swab | NA | NA | N | NA | NA | NA | 1 | Sabin | 5.6 | 2 | 10 | 13 | 18 | 4 | 10 | 16 | 18 |
| | | | | | | | | | | | | | 2 | Sabin | 5.7 | 3 | 10 | 8 | 10 | 5 | 10 | 9 | 10 |
| | | | | | | | | | | | | | 3 | Sabin | 5.9 | 0 | 11 | 8 | 19 | 1 | 11 | 10 | 19 |
| Dardanoni et al, 1962 | swab | 0 | MK, HeLa | NA | yes[l] | - | - | - | N | 1:2 or 1:4 | <2–≥2 or <4–≥4 | 1, 2, 4, 6, 8.5 wk | 1 | Sabin | 6–6.9[h] | - | - | - | - | 13 | 22 | 10 | 13 |
| Ingram et al, 1962[m] | stool | 0–7 (2x) | MK | NA | NA | stool | 2x per wk for 6–8 wk | yes | MI | 1:4 | 4-fold rise | 2–4 wk | 1 | Sabin | 6 | 9 | 10 | 14 | 14 | - | - | - | - |
| Paul et al, 1962[n] | swab | 0–7 (2x) | MK, HEp-2 | NA | NA | swab | 2x per wk for ≥4 wk | NA | N | 1:4 | <4–≥4 | 1 mo | 1 | Lederle | 5–5.5[h] | 6 | 20 | 17 | 28 | 5 | 20 | 12 | 28 |
| | | | | | | | | | | | | | 2 | Lederle | 5–5.5[h] | 3 | 14 | 2 | 21 | 3 | 14 | 2 | 21 |
| | | | | | | | | | | | | | 3 | Lederle | 5–5.5[h] | 7 | 9 | 22 | 25 | 6 | 9 | 21 | 25 |

| | | | | | | | | | | | | | | | | | | | | | | | |
|---|---|---|---|---|---|---|---|---|---|---|---|---|---|---|---|---|---|---|---|---|---|---|---|
| Urasawa, 1964[o] | stool | 0 | HeLa | NA | NA | - | - | - | N | 1:4 | 4-fold rise | 1 mo | 1 | Sabin | 4.8–5.7 | - | - | - | - | 13 | 20 | 39 | 60 |
| | | | | | | | | | | | | | 2 | Sabin | 4.5–5.8 | - | - | - | - | 16 | 21 | 46 | 67 |
| | | | | | | | | | | | | | 3 | Sabin | 5.3–6.3 | - | - | - | - | 15 | 20 | 47 | 70 |
| Spano et al, 1965 | stool/ swab | 0 | MK, HeLa | NA | yes[l] | stool/ swab | 15 d | no | - | - | - | - | 1 | NA | NA | 3 | 14 | 3 | 19 | - | - | - | - |
| | | | | | | | | | | | | | 2 | NA | NA | 3 | 14 | 5 | 19 | - | - | - | - |
| | | | | | | | | | | | | | 3 | NA | NA | 1 | 8 | 7 | 22 | - | - | - | - |
| JLPRC, 1966[p] | stool | NA (weekly) | MK | NA | NA | stool | 2, 4 wk | no | N | 1:4 | <4–≥4 | 4, 8 wk | 1 | Sabin | 5.5[h] | 6 | 25 | 196 | 430 | 10 | 14 | 193 | 211 |
| | | | | | | | | | | | | | 2 | Sabin | 5.5[h] | 9 | 56 | 99 | 321 | 34 | 50 | 130 | 149 |
| | | | | | | | | | | | | | 3 | Sabin | 5.5[h] | 7 | 21 | 129 | 354 | 25 | 34 | 151 | 180 |
| Nardi et al, 1966[q] | stool | 1–2 | MK, HA | NA | NA | stool | 4, 7, 14, 21, 28 d | yes | N | 1:4 | <4–≥4 or 4-fold rise | 45–60 d | 1 | Sabin | 5.5 | 2 | 14 | 1 | 26 | 28 | 48 | 27 | 54 |
| | | | | | | | | | | | | | 2 | Sabin | 5.5 | 7 | 14 | 8 | 26 | 29 | 36 | 34 | 53 |
| | | | | | | | | | | | | | 3 | Sabin | 5.5 | 7 | 14 | 8 | 26 | 27 | 49 | 28 | 64 |
| Ramos-Alvarez, 1966, mOPV[r] | swab | 0 | HK | NA | NA | NA | NA | NA | N | undiluted | negative to positive in undiluted serum | 4–5 wk | 1 | Sabin | 5.5 | 29 | 64 | 31 | 39 | 38 | 64 | 37 | 39 |
| | | | | | | | | | | | | | 2 | Sabin | 5.5 | 23 | 44 | 37 | 43 | 26 | 44 | 41 | 43 |
| | | | | | | | | | | | | | 3 | Sabin | 5.5 | 26 | 62 | 25 | 29 | 31 | 62 | 28 | 29 |
| Ramos-Alvarez, 1966, tOPV[r] | swab | 0 | HK | NA | NA | - | - | - | N | undiluted | negative to positive in undiluted serum | 7–8 wk | 1 | Sabin | 5.7 | - | - | - | - | 31 | 45 | 57 | 69 |
| | | | | | | | | | | | | | 2 | Sabin | 5–5.3 | - | - | - | - | 36 | 52 | 62 | 64 |
| | | | | | | | | | | | | | 3 | Sabin | 5.4 | - | - | - | - | 20 | 40 | 43 | 87 |
| Sureau et al, 1966 | stool | 2 | KB | yes | NA | stool | 8 d | no | - | - | - | - | 1 | NA | NA | 4 | 19 | 5 | 18 | - | - | - | - |
| | | | | | | | | | | | | | 2 | NA | NA | 3 | 19 | 5 | 18 | - | - | - | - |
| | | | | | | | | | | | | | 3 | NA | NA | 2 | 19 | 5 | 18 | - | - | - | - |
| John & Christopher, 1975[s] | stool/ swab | 0 | MK, HEp-2 | yes | NA | stool/ swab | 1, 2 wk | yes | MN | 1:10 | <10–≥10 | 8 wk | 1 | Sabin | 6[h] | - | - | - | - | 10 | 40 | 4 | 23 |
| | | | | | | | | | | | | | 2 | Sabin | 5[h] | - | - | - | - | 31 | 44 | 10 | 26 |
| | | | | | | | | | | | | | 3 | Sabin | 5.5[h] | - | - | - | - | 13 | 50 | 7 | 26 |
| Faden et al, 1992 | stool | 0 | MK, HEp-2 | no | yes[t] | - | - | - | MN | 1:10 | >10 | 1 mo | 1 | Sabin | 5.5–6.4 | - | - | - | - | - | - | - | - |
| | | | | | | | | | | | | | 2 | Sabin | 4.5–5.5 | - | - | - | - | - | - | - | - |
| | | | | | | | | | | | | | 3 | Sabin | 5.2–6.2 | - | - | - | - | - | - | - | - |

| | | | | | | | | | | | | | | | | | | | | | | | |
|---|---|---|---|---|---|---|---|---|---|---|---|---|---|---|---|---|---|---|---|---|---|---|---|
| Kok et al, 1992 | stool | 0 | NA | NA | NA | - | - | - | MN | 1:8 | ≥8 after 3 doses | 2 mo | 1 | Sabin | 6[h] | - | - | - | - | - | - | - | - |
| | | | | | | | | | | | | | 2 | Sabin | 5[h] | - | - | - | - | - | - | - | - |
| | | | | | | | | | | | | | 3 | Sabin | 5.5[h] | - | - | - | - | - | - | - | - |
| Maldonado et al, 1997 | stool | 7, 0, +7 | PMK, BGMK, RD, MRC-5 | NA | yes[u] | - | - | - | MN | 1:8 | <8–≥8 or 4-fold increase over expected residual maternal antibody | 8 wk | 1 | Sabin | 6[h] | - | - | - | - | - | - | - | - |
| | | | | | | | | | | | | | 2 | Sabin | 5[h] | - | - | - | - | - | - | - | - |
| | | | | | | | | | | | | | 3 | Sabin | 5.6[h] | - | - | - | - | - | - | - | - |
| Triki et al, 1997 | stool | 0 | HEp-2C, RD | NA | yes[v] | - | - | - | MN | 1:4 | <8–≥8 or 4-fold rise | 1 mo | 1 | Sabin | 6[h] | - | - | - | - | - | - | - | - |
| | | | | | | | | | | | | | 2 | Sabin | 5[h] | - | - | - | - | - | - | - | - |
| | | | | | | | | | | | | | 3 | Sabin | 5.8[h] | - | - | - | - | - | - | - | - |

Abbreviations: 2x: two samples; 3x: three samples; IR: infected responders; IT: infected total; JLPRC: Japan Live Poliovaccine Research Commission; MI: metabolic inhibition assay; MN: microneutralization assay; mOPV, monovalent oral poliovirus vaccine; N: neutralization assay; NA: not reported; TCID: tissue culture infective dose; tOPV, trivalent oral poliovirus vaccine; UR: uninfected responders; UT: uninfected total.

[a] See Table 1 for citation details.
[b] Studies reporting the use of rectal swabs are indicated as ‘swab’; all other fecal sampling methods are listed as ‘stool’.
[c] Studies reporting the collection of fecal specimens prior to or at the time of vaccine delivery, without further details, are listed here as being obtained on the day of vaccine delivery.
[d] ‘Multiple’ refers to the combining of data across more than one postvaccination fecal samples during the assessment of vaccine take.
[e] Antibody titers are expressed as the reciprocal of the dilution producing virus neutralization during neutralization tests.
[f] Data were summed across individuals infected with Coxsackie A, Coxsackie B, and ECHO/other viruses; no enterovirus coinfections were referred to by the authors.
[g] Dose-specific take data from this study were not included in the meta-analysis as the age range of nonpolio enterovirus (NPEV)-infected individuals (1−4 months) did not match that of uninfected individuals (3−4 months); the younger age of infected individuals may therefore be responsible for the differences observed between these groups.
[h] Testing of vaccine potency described in report.
[i] Additional details were obtained from a report by Voroshilova et al [1].
[j] Suckling and adult cotton rats were also tested during virus isolations.
[k] The total number of uninfected individuals was not reported directly by the authors; however, 33 children under 4 years of age (the age range in which the influence of NPEVs on oral poliovirus vaccine [OPV] take was examined) lacked any virus during the prevaccination survey; this number was therefore used as the total number of uninfected controls during the meta-analysis. Prevaccination samples were mixed before viral isolation in this study, as were postvaccination samples obtained on 3 and 5 days, 12 and 15 days, and 22 and 24 days.
[l] Hemagglutination/hemagglutination-inhibition assay.
[m] Any poliovirus excretion within the 4-week period after vaccine delivery (rather than the full study period of 10 weeks) was considered as indicative of vaccine take.
[n] This study was not included in the primary analysis as 8 individuals excreting type 3 poliovirus at the time of vaccination were not excluded from the data.
[o] Methods adopted by Urasawa [2] were assumed to follow those described in a prior publication by the same author [3].
[p] The study reported outcomes relative to the presence or absence of NPEVs for vaccine take at 2 and 4 weeks after OPV delivery, and seroconversion 4 and 8 weeks after vaccination; in both cases, the 4-week data were available for more individuals, and were therefore used in the meta-analysis. Stool samples were collected immediately prior to vaccination, then weekly throughout the study; the reporting of concurrent NPEVs included infections before vaccination for OPV take data, and infections immediately before or

after vaccination for seroconversion data. The numbers of responders reported here are inferred from proportions presented in the published article, rounded to the nearest whole number.

[q] Data regarding vaccine take were obtained from the report by Monaci et al [4].

[r] Additional details were obtained from a report by Sabin [5].

[s] Additional details were obtained from reports by John and Jayabal [6] and John [7]. The presented data were summed across ditypic and tritypic seronegative individuals; data for monotypic seronegative individuals were not reported according to poliovirus serotype. The numbers of responders reported here are inferred from proportions presented in the published article, rounded to the nearest whole number.

[t] Virus isolations from nasopharyngeal secretions were also performed in this study. Hemadsorption was used for the detection of influenza and parainfluenza; samples demonstrating cytopathic effects or hemadsorption were tested using an immunofluorescence assay for respiratory viruses. Electron microscopy was used for the detection of rotavirus and adenovirus in fecal samples.

[u] The presence of enteroviruses was confirmed by RNA−RNA hybridization, and the presence of adenovirus 40/41, astrovirus, and rotavirus infections was examined using screening immunoassays.

[v] The presence of rotavirus and adenovirus type 40/41 was tested by ELISA.

**Supplementary Table 2. Subgroup and Sensitivity Analyses**

| | Serological Response, OR (95% CI) | | | | | Vaccine Take, OR (95% CI) | | | | |
|---|---|---|---|---|---|---|---|---|---|---|
| | No. Studies | Type 1 | Type 2 | Type 3 | Overall | No. Studies | Type 1 | Type 2 | Type 3 | Overall |
| Baseline[a] | 9 | 0.44 (0.23−0.84) | 0.53 (0.19−1.46) | 0.56 (0.27−1.12) | 0.47 (0.20−1.04) | 9 | 0.50 (0.28−0.89) | 0.59 (0.30−1.13) | 0.67 (0.26−1.71) | 0.58 (0.26−1.24) |
| Stratification by Vaccine Formulation: | | | | | | | | | | |
| Monovalent OPV | 4 | 0.17 (0.07−0.44) | 0.15 (0.03−0.66) | 0.18 (0.05−0.66) | 0.17 (0.07−0.42) | 5 | 0.31 (0.15−0.64) | 0.31 (0.13−0.70) | 0.25 (0.06−1.04) | 0.30 (0.13−0.69) |
| Trivalent OPV | 5 | 0.73 (0.38−1.41) | 1.06 (0.37−3.03) | 0.87 (0.39−1.94) | 0.92 (0.45−1.86) | 4 | 0.79 (0.37−1.68) | 1.07 (0.48−2.38) | 1.31 (0.41−4.15) | 1.11 (0.48−2.63) |
| Stratification by Income Group: | | | | | | | | | | |
| Low-, Lower-Middle or Upper-Middle-income | 5 | 0.31 (0.13−0.72) | 0.33 (0.08−1.39) | 0.34 (0.14−0.86) | 0.31 (0.11−0.82) | 4 | 0.32 (0.16−0.64) | 0.31 (0.13−0.78) | 0.28 (0.09−0.86) | 0.29 (0.12−0.63) |
| High-income | 4 | 0.68 (0.27−1.70) | 0.99 (0.17−5.68) | 1.07 (0.37−3.07) | 0.80 (0.26−2.42) | 5 | 0.83 (0.38−1.84) | 1.09 (0.44−2.71) | 1.91 (0.57−6.39) | 1.17 (0.53−2.56) |
| Sensitivity Analysis: | | | | | | | | | | |
| Enterovirus Coinfection (Including Some Poliovirus)[b] | 15 | 0.51 (0.34−0.75) | 0.67 (0.33−1.36) | 0.58 (0.33−1.02) | 0.55 (0.33−0.89) | 10 | 0.44 (0.25−0.77) | 0.65 (0.34−1.25) | 0.67 (0.26−1.71) | 0.55 (0.27−1.10) |
| n > 5 in Infected/Control Group[c] | 9 | 0.44 (0.23−0.84) | 0.53 (0.19−1.46) | 0.56 (0.27−1.12) | 0.47 (0.20−1.04) | 10 | 0.48 (0.28−0.81) | 0.59 (0.30−1.13) | 0.63 (0.26−1.50) | 0.54 (0.26−1.08) |

Abbreviations: CI, confidence interval; OPV, oral poliovirus vaccine; OR, odds ratio.

[a] Baseline data correspond with those presented in Figures 2 and 4.

[b] During meta-regression, effect size for per-dose seroconversion was not significantly influenced by poliovirus serotype (likelihood ratio test [LRT], *P* = .434), formulation (mono-, bi- or tri-valent OPV; LRT, *P* = .248), or trial setting (low-, lower-middle-, or upple-middle- vs high-income countries; LRT, *P* = .303). Effect size for per-dose take was significantly influenced by trial setting (LRT, *P* = .013), but not poliovirus serotype (LRT, *P* = .325) or formulation (mono- vs tri-valent OPV; LRT, *P* = .054).

[c] During meta-regression, effect size for per-dose take was significantly influenced by formulation (LRT, *P* = .018) and trial setting (LRT, *P* = .035), but not poliovirus serotype (LRT, *P* = .557). Meta-regression outcomes for per-dose seroconversion were not affected.

**Supplementary Table 3. Summary of Studies Examining the Influence of Diarrhea on Serological Response and/or Take Following Oral Poliovirus Vaccine Delivery**

| | | Take Measurement | | | Seroconversion Measurement | | | | | | | Take Data | | | | Per-dose Seroconversion Data | | | | Overall Seroconversion Data | | | |
|---|---|---|---|---|---|---|---|---|---|---|---|---|---|---|---|---|---|---|---|---|---|---|---|
| Study[a] | Definition of Diarrhea | Sample | Timing | Multiple[b] | Test | Lowest Dilution tested | Seroconversion Criteria[c] | Timing | Type | Strain | Dose (log10 TCID50) | DR | DT | NR | NT | DR | DT | NR | NT | DR | DT | NR | NT |
| Mahmoud et al, 1976 | NA | stool | 7, 14, 21 d | yes | - | - | - | - | NA | NA | NA | 1 | 10 | 9 | 14 | - | - | - | - | - | - | - | - |
| WHO, 1995[d] | ≥4 watery stools in 24 h preceding OPV delivery | - | - | - | MN | 1:8 | <8–≥8 or 4-fold rise over expected residual maternal antibody | >4 wk | 1 | Sabin | 6–6.3[e] | - | - | - | - | - | - | - | - | 151 | 177 | 420 | 502 |
| | | | | | | | | | 2 | Sabin | 4.7–5[e] | - | - | - | - | - | - | - | - | 164 | 177 | 484 | 502 |
| | | | | | | | | | 3 | Sabin | 5.5–5.8[e] | - | - | - | - | - | - | - | - | 113 | 177 | 377 | 502 |
| Myaux et al, 1996[f] | ≥3 watery stools per day for <7 days, and absence of other complications | - | - | - | MN | 1:16 | <16–≥16 or 4-fold rise over expected residual maternal antibody | 4 wk | 1 | Sabin | 6[e] | - | - | - | - | 33 | 99 | 71 | 211 | - | - | - | - |
| | | | | | | | | | 2 | Sabin | 5[e] | - | - | - | - | 40 | 98 | 116 | 209 | - | - | - | - |
| | | | | | | | | | 3 | Sabin | 5.5[e] | - | - | - | - | 27 | 99 | 88 | 212 | - | - | - | - |
| Posey et al, 1997[d] | ≥3 watery stools in a 24 h period in the 2 wk preceding OPV delivery[g] | - | - | - | MN | 1:8 | <8–≥8 or 4-fold rise over expected residual maternal antibody | 4–6 wk | 1 | Sabin | 6–6.3[e] | - | - | - | - | 11 | 32 | 196 | 490 | 72 | 84 | 559 | 644 |
| | | | | | | | | | 2 | Sabin | 4.7–5[e] | - | - | - | - | 7 | 22 | 162 | 305 | 73 | 84 | 601 | 644 |
| | | | | | | | | | 3 | Sabin | 5.5–5.8[e] | - | - | - | - | 4 | 36 | 186 | 598 | 45 | 84 | 423 | 644 |

Abbreviations: DR: diarrheal responders; DT: diarrheal total; MN: microneutralization assay; NA: not reported; NR: nondiarrheal responders; NT: nondiarrheal total; OPV, oral poliovirus vaccine; TCID: tissue culture infective dose; WHO, World Health Organization.

[a] See Table 1 for citation details.
[b] 'Multiple' refers to the combining of data across more than one postvaccination fecal samples during the assessment of vaccine take.
[c] Antibody titers are expressed as the reciprocal of the dilution producing virus neutralization during neutralization tests.
[d] The reports by the WHO [8] and Posey et al [9] describe trials conducted in Brazil and the Gambia in which 4 different formulations of trivalent OPV (with varying potencies) were randomly assigned to vaccinees. The effect of diarrhea was observed to be comparable among the formulations, and data were therefore pooled across vaccine groups. Both trials are described in the report by the WHO [8], with additional dose-specific data documented by Posey et al [9] for the trial conducted in Brazil. For the study by Posey et al [9], seroconversion rates for the OPV dose delivered at 6 weeks of age were used in this analysis; these data were available for the greatest number of infants. Diarrhea-associated interference was also apparent for doses delivered at 10 and 14 weeks in this study, although the effect size was smaller.
[e] Testing of vaccine potency described in report.
[f] Infants were treated with oral rehydration therapy after enrollment.
[g] In the report on this trial by the WHO [8], which was used during the analysis of diarrhea-associated interference over multiple doses of trivalent OPV (Figure 6), diarrhea was defined as ≥4 watery stools per day for at least 3 consecutive days in the 2 weeks preceding OPV delivery.

## References


1. Voroshilova MK TG, Tolskaya EA, Balayan MS, Markova A. Antibody response in children vaccinated with trivalent live vaccine from A. B. Sabin's strains. In: Oral Live Poliovirus Vaccine: Papers presented at the IVth Scientific Conference of the Institute of Poliomyelitis and Virus Encephalitis and the International Symposium on Live Poliovirus Vaccine, **1960**:317–30.
2. Urasawa S. Studies on the interference between group B coxsackie viruses and polioviruses. 2. Comparisons of the effects of coxsackie B 5 virus on the immunizations with mono- and polyvalent live poliovirus vaccines. Sapporo Igaku Zasshi **1964**; 26:8–14.
3. Urasawa S. Studies on the interference between group B coxsackie viruses and polioviruses. I. The interference with attenuated polioviruses by coxsackie B5 virus in man. Sapporo Igaku Zasshi **1964**; 25:247–57.
4. Monaci V, Vitali MA, Nardi G, Bellelli E, Trombetta N, Bassanetti F. Revaccination with Sabin's attenuated trivalent oral polio vaccine in some areas of northern Italy. Ann Sclavo **1965**; 7:341–58.
5. Sabin AB. Vaccination against poliomyelitis in economically underdeveloped countries. Bull World Health Organ **1980**; 58:141–57.
6. John TJ, Jayabal P. Oral polio vaccination of children in the tropics. I. The poor seroconversion rates and the absence of viral interference. Am J Epidemiol **1972**; 96:263–9.
7. John TJ. Oral polio vaccination of children in the tropics. II. Antibody response in relation to vaccine virus infection. Am J Epidemiol **1975**; 102:414–21.
8. WHO. Factors affecting the immunogenicity of oral poliovirus vaccine: a prospective evaluation in Brazil and the Gambia. J Infect Dis **1995**; 171:1097–106.
9. Posey DL, Linkins RW, Oliveria MJ, Monteiro D, Patriarca PA. The effect of diarrhea on oral poliovirus vaccine failure in Brazil. J Infect Dis **1997**; 175:S258–63.
